# Assessment of safety and feasibility of a new technical variant of gastropexy for percutaneous endoscopic gastrostomy: an experience with 435 cases

**DOI:** 10.1186/1471-230X-9-48

**Published:** 2009-06-26

**Authors:** Paulo MO Campoli, Daniela MM Cardoso, Marília D Turchi, Flávio H Ejima, Orlando M Mota

**Affiliations:** 1 Department of Digestive Endoscopy, Araújo Jorge Hospital, Goiás Anticancer Association, Goiânia, GO, Brazil; 2 Department of Gastrointestinal Oncology, Araújo Jorge Hospital, Goiás Anticancer Association, Goiânia, GO, Brazil; 3 Department of Community Health, Institute of Tropical Pathology and Public Health, Federal University of Goiás, Goiânia, GO, Brazil

## Abstract

**Background:**

Percutaneous Endoscopic Gastrostomy (PEG) performed through the Introducer Technique is associated with lower risk of surgical infection when compared to the Pull Technique. Its use is less widespread as the fixation of the stomach to the abdominal wall is a stage of the procedure that is difficult to be performed. We present a new technical variant of gastropexy which is fast and easy to be performed. The aim of this study was to evaluate the safety and feasibility of a new technical variant of gastropexy in patients submitted to gastrostomy performed through the Introducer Technique.

**Methods:**

All the patients submitted to PEG through the Introducer Technique were evaluated using a new technical variant of gastropexy, which consists of two parallel stitches of trasfixation sutures involving the abdominal wall and the gastric wall, performed with a long curved needle.

Prophylactic antibiotics were not used. Demographic aspects, initial diagnosis, indication, sedation doses, morbidity and surgical mortality were all analyzed.

**Results:**

Four hundred and thirty-five consecutive PEGs performed between June 2004 and May 2007 were studied. Nearly all the cases consisted of patients presenting malignant neoplasia, 79.5% of which sited in the head and neck. The main indication of PEG was dysphagia, found in 346 patients (79.5%). There were 12 complications (2.8%) in 11 patients, from which only one patient had peristomal infection (0.2%). There was one death related to the procedure.

**Conclusion:**

Gastropexy with the technical variant described here is easy to be performed and was feasible and safe in the present study. PEG performed by the Introducer Technique with this type of gastropexy was associated with low rates of wound infection even without the use of prophylactic antibiotics.

## Background

Percutaneous Endoscopic Gastrostomy (PEG), described in 1980 [1,2], has replaced Conventional Surgical Gastrostomy as it has proved to be more advantageous. Its use, therefore, has grown rapidly in daily clinical practice [3].

Several technical variants have been described for performing PEG, with the one proposed by Gauderer et al [1] topping the list in the majority of centers. Known as the Pull Technique, it is easy to be performed and quite safe. Through this technique, the gastric tube (G-tube) is pulled through the mouth and the esophagus, which results in an increased risk of peristomal infection [4,5], despite the routine use of antibiotic prophylaxis, as is the risk of tumoral implantation in the surgical wound in patients presenting malignant tumors [6].

There is a technical variant, named the Introducer Technique, in which the G-tube is introduced by means of percutaneous punction in an attempt to avoid its passage through the mouth. It can be performed under radiological [7] or endoscopic [2,8-13] guidance and also offers the great advantage of low risk of peristomal infection, which renders the use of prophylactic antibiotics unnecessary [7,8,14]. This technique is also associated with low risk of tumor wound implantation [15]. A lower risk of infection and lower risk of tumor implantation has motivated several authors to use the Introducer Technique instead of using the Pull Technique for PEG [4,6,8,15,16].

The Introducer Technique almost always involves a stage in which the stomach is fixated to the abdominal wall (gastropexy). For such fixation, T-fasteners [7,16,17], Fogarty catheters [18] or stitches [2,5,8-11,14,19,20] can be used. The use of stitches was first described by Hashiba in 1980 [2]. In 1999, Kiser et al [10] reported gastropexy performed with two straight needles, a method used by us until June 2004 [8]. Several authors [5,9,11,14,20] have recently described the use of a device that also contains two straight needles for the easier performance of gastropexy.

We have recently published a successful series of 142 cases [8] of PEGs with an Introducer Technique variant which employs stitches with straight needles in order to fixate the anterior gastric wall to the abdominal wall, followed by the introduction of a G-tube by means of a percutaneous punction.

The present study describes a new technical variant of gastropexy which uses a long curved needle. It aims to investigate the feasibility and safety of the procedure.

## Methods

### Patients

We studied all patients referred to perform PEG in a tertiary cancer hospital between June 2004 and May 2007.

Exclusion criteria comprised patients with Body Mass Index (BMI) ≥ 30 kg/m^2^, those on whom PEG was performed without gastropexy once the stomach was adequately fixated to the abdominal wall as well as those on whom PEG could not be performed.

Almost all the procedures were performed in the endoscopy room, with patients under conscious sedation and monitored by a pulse oximeter. Supplementary oxygen was used when necessary. Olympus GIF-V video gastroscope and Olympus CV-100 video processor were used (Olympus America Inc., Melville, New York, USA). All the procedures were performed by three authors specialized in digestive endoscopy and with experience in interventional endoscopic techniques.

Endoscopic dilation was attempted when stenosis was present and whenever possible performed with Eder-Puestow dilators. Prophylactic antibiotics were not used. All the patients were fed through the G-tube on the same day of the procedure.

An informed consent was obtained from all patients and this study was approved by the Ethical Institutional Review Board.

### Suture method

**Figure 1:**
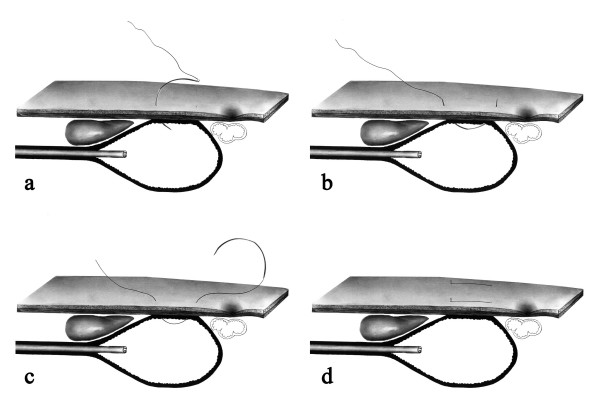
**Suture method**. Transfixation suture with curved needle involving the abdominal and the gastric wall, performed under endoscopic guidance (Figures 1a, b and 1c). A second transfixation U-shaped stitch was employed in parallel with the first one (Figure 1d).

Following a thorough endoscopic examination, the patient was placed in the supine position with upper limbs restraint. The insertion point was identified by transillumination and palpation of the abdominal wall. By using an aseptic technique along with lidocaine-induced local anesthesia, a stitch was employed involving the abdominal wall and the anterior gastric wall under endoscopic guidance (Figures 1a, b and 1c). A 7.6 cm-long needle of 1/2 circle curvature and polypropylene thread was used (B. Braun Medical Products, Aesculap Division, Tuttlingen, Germany). This same procedure was repeated and another U-shaped stitch was used parallel to the first stitch (Figure 1d). These two stitches provided the fixation of the anterior stomach wall on the abdominal wall.

### Gastric tube introduction technique

#### Abdominal Wall Path

**Figure 2:**
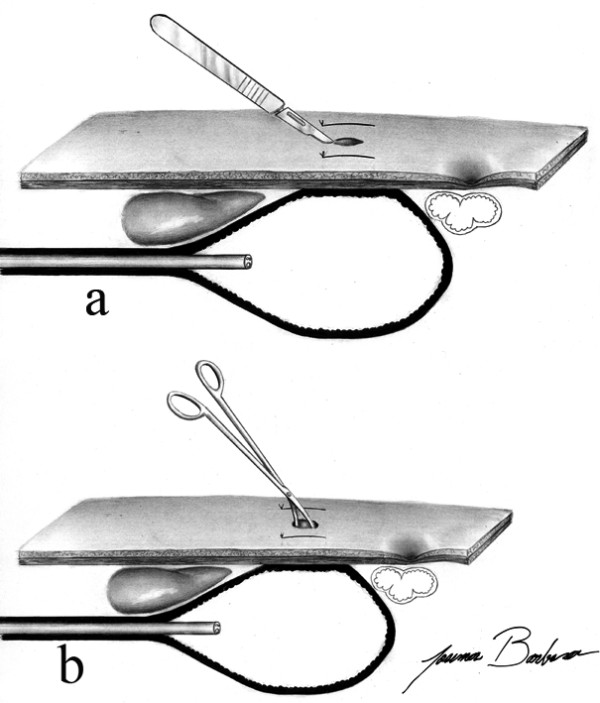
**Gastric tube introduction technique – abdominal wall path**. A cutaneous incision was made between the two stitches (Figure. 2a) and afterwards a path was made through the abdominal wall by using Metzenbaum scissors without puncturing the gastric wall (Figure. 2b).

A cutaneous incision between the two stitches was performed, under local anesthesia with lidocaine (Figure 2a) and a tissue dissection with surgical scissors was made in order to reach the gastric wall without perforate it (Figure 2b).

#### Trocar Puncture

**Figure 3:**
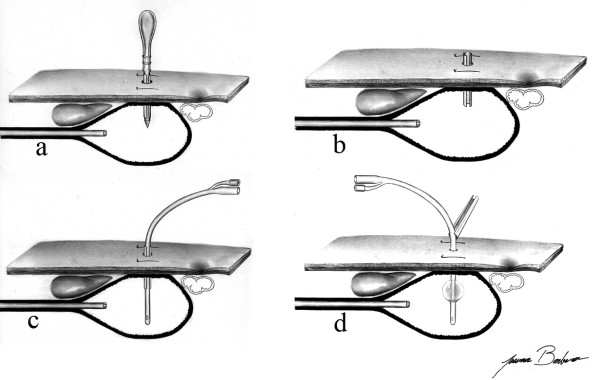
**Gastric tube introduction technique – trocar puncture and gastric tube introduction**. The gastric wall was punctured with a trocar introducer with a peel-away sheath (Figure. 3a and 3b), the G-tube was introduced through the sheath (Figure. 3c), the balloon was then inflated and the sheath was removed (Figure. 3d).

A metal trocar proper designed for PEG was used. A trocar puncture was performed through the path in order to reach the gastric cavity (Figure 3a). The trocar was removed and an external metal sheath with a longitudinal fenestration stayed in the path (Figure 3b).

#### Gastric Tube Introduction

A G-tube (16 Fr) was introduced through the sheath (Figure 3c) and the balloon was inflated (Figure 3d). The sheath was removed and disconnected from the G-tube through the longitudinal fenestration (Figure 3d).

### Video

Watch the video containing the described procedure. [see Additional file 1].

### Follow up

The patients received daily dry dressing and the gastropexy stitches were removed between postoperative days 10 and 12. The G-tube removal or changing was performed whenever needed. Wound infection evaluation was provided in all cases.

### Analyzed parameters

The feasibility of the procedure was evaluated through the percentage of success in the performance of gastropexy among the cases included in the study.

To evaluate the safety of the method the complications were classified into two categories: minor and major complications. Minor complications were the ones which occurred during the procedure and were solved with no need of additional intervention. The major complications needed additional interventions or added risk to the patients. The safety was also evaluated by procedure related mortality.

## Results

### Patients' profile

During 36 months 515 patients were referred to the Endoscopy Unit to have PEG and 44 were excluded from the present study. The main reason for not performing this procedure was non dilatable stenosis (Table 1).

**Table 1:** Exclusion criteria from the present study of 44 patients referred to the Endoscopy Unit to perform PEG*.

Causes	number	%
BMI** ≥ 30 kg/m^2^	3	6.8
**PEGs suture-free technique**	6	13.6
**PEGs could not be performed**		
Non dilatable stenosis	26	59.1
Neoplasias affecting stomach	3	6.8
Gastric ulcer perforation	2	4.5
Patients with ascites	2	4.5
Partial gastrectomy	1	2.3
Respiratory failure associated to supine position	1	2.3

*PEG, Percutaneous Endoscopic Gastrostomy

**BMI, Body Mass Index

**Figure 4:**
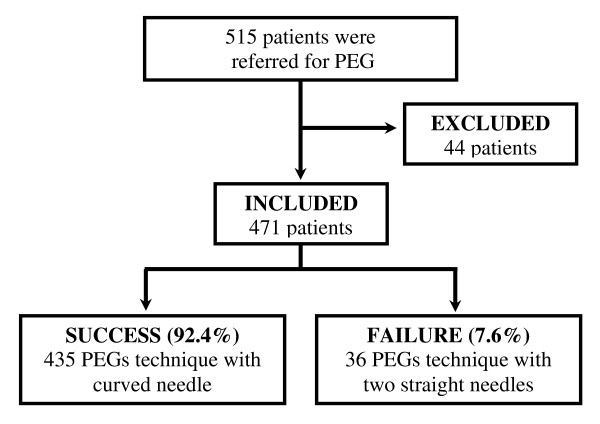
**Distribution of patients referred for PEG**.

Among the 435 patients where curved needle gastropexy was performed (Figure 4), a clear predominance of the male gender was observed (4.4:1) and the mean age was 58.8 (8 – 99 years old). The vast majority of patients had malignant neoplasias with predominance of head and neck tumors (79.5%), followed by esophagus tumors (17.0%) and lung tumors (2.1%). Only six patients presented neurological disorders (Table 2). The main indication for the procedure was dysphagia in 346 patients (79.5%), followed by other indications listed in Table 2.

**Table 2:** Clinical features and morbimortality of 435 patients submitted to PEG* with curved needle.

Variable	number	%
**Gender**		
Male	354	81.4
Female	81	18.6

**Baseline disease**		
Head/Neck neoplasia	346	79.5
Esophagus neoplasia	74	17.0
Lung neoplasia	9	2.1
Neurologic disease	6	1.4

**Indication**		
Dysphagia	346	79.5
Preoperative	57	13.1
Salivary fistula	22	5.1
Nasal regurgitation	10	2.3

**Minor complications**		
Bleeding	4	0.9
Respiratory failure	3	0.7

**Major complications**		
Pneumoperitoneum	2	0.5
Leakage	2	0.5
Wound infection	1	0.2

**Mortality**	1	0.2

*PEG, Percutaneous Endoscopic Gastrostomy

In four patients the procedure had to be performed under general anesthesia in the surgery room. In the other patients the PEG was performed in the endoscopy room and the conscious sedation was obtained with doses of midazolam ranging from 0 to 13 mg with a median of 4 mg (interquartile range, 3–5) associated or not with doses between 0 and 130 mg with a median of 40 mg (interquartile range, 30–50) of meperidine.

In 37 patients peptic ulcer was diagnosed (gastric or duodenal). Successful endoscopic dilation was performed in 24 patients. Nine patients were diagnosed as having a second synchronous neoplasia during the performance of PEG. Four patients had tracheoesophageal fistula. Two patients had previous partial gastrectomy.

### Feasibility Evaluation

Among the 471 patients included, gastropexy was not performed in 36 of them through the method described in this study as the curved needles were unable to reach the gastric cavity due to excessively thick abdominal walls. In this group of patients, gastropexy was performed with two straight needles.

The remaining four hundred and thirty five suture-based PEGs were performed with the new curved-needle method described, representing a success index of 92.4% (Figure 4).

### Safety Evaluation

Among the 435 patients in whom gastropexy was performed with a curved needle, morbidity consisted of 12 events (2.8%) in 11 patients. Minor complications occurred in 7 patients and consisted of four cases of gastric wall bleeding which were observed during the procedure and controlled with local measures and three cases of respiratory failure controlled with the habitual measures of ventilatory assistance and the use of naloxone or flumazenil.

Five major complications occurred in four patients. Section of the gastric wall caused by the thread of the first stitch occurred in one patient and resulted in pneumoperitoneum. Laparotomy was necessary to conclude the gastrostomy. The second patient started with abdominal pain on the postoperative period and a large pneumoperitoneum was identified. This patient underwent surgery with no other findings. The third patient evolved with a gastro-cutaneous fistula closed after changing the G-tube for a Dobbhoff tube. The fourth patient presented wound infection (0.2%) on the first postoperative week. This patient received oral antibiotic with good outcome and resolution of the infection. This same patient developed wound leakage on postoperative day 50 due to severe malnutrition and cancer cachexia and died. There were one procedure-related death (0.2%), as described above.

## Discussion

This study presents a high success rate of a simple and safe technical variant of the gastropexy during PEG, in patients with malignant diseases. Moreover, in this study this procedure was associated with a low surgical infection rate.

The low wound infection rate is the great advantage of the Introducer Technique. Pull Technique PEG performed with antibiotic prophylaxis has wound infection rates around 8% [21,22]. On the other hand, the series already published which used the Introducer Technique are presented in Table 3 and the pooled of available studies shows an infection rate of 1.4% (ranging from 0 to 3.6%).

**Table 3:** Published series of PEGs by the Introducer Technique

Author [ref]	Year	Gastropexy	Antibiotics	N	Infection (N)	Infection (%)
Russell TR [12]	1984	No	N/A	28	1	3.6
Hashiba K [19]	1987	Suture	N/A	56	0	0.0
Kadota T [13]	1991	No	N/A	89	3	3.4
Robertson FM [18]	1996	Fogarty	Yes	20	0	0.0
Tucker AT [16]	2003	T-fastener	Yes	29	0	0.0
Maetani I [4]	2003	No	Yes	29	0	0.0
Dormann AJ [9]	2006	Suture	Yes	46	1	2.2
Saito M [11]	2007	Suture	N/A	82	0	0.0
Campoli PMO [8]	2007	Suture	No	142	4	2.8
Toyama Y [20]	2007	Suture	Yes	30	1	3.3
Foster JM [17]	2007	T-fastener	No	149	5	3.4
Shastri YM [14]	2008	Suture	Yes	47	1	2.1
Shastri YM [14]	2008	Suture	No	46	1	2.2
Horiuchi A [5]	2008	Suture	Yes	68	0	0.0
Current series	2008	Suture	No	435	1	0.2

**Pooled**				**1,296**	**18**	**1.4** **[95%CI: 0.9–2.2]**

N/A, information not available

CI, Confidence Interval

The cases presented here were performed using this Introducer Technique, and even without using the prophylactic antibiotics, the peristomal infection rate was as low as 0.2%.

There are few studies comparing Pull Technique and Introducer Technique.

Three non-randomized studies with a small number of cases have compared the Pull Technique with the Introducer Technique. Deitel et al [23] reported that the Introducer Technique was not associated with peristomal infection, whereas Tucker et al [16] concluded that the risk of complications was significantly lower with this technique. The third study published recently showed that the Introducer Technique was associated with lower risk of peristomal infection, lower risk of aspiration pneumonia and lower postoperative hospital stay [20].

Two studies that compared the two techniques through a prospective and randomized trials were lead by Maetani et al [4] and Horiuchi et al [5]. They found that the risk of peristomal infection was lower when the Introducer Technique was used.

In the present study major and minor complications occurred in a small number of cases with few repercussions for patients, yielding a morbidity rate of 2.8% and an acceptable mortality rate of 0.2%. We have a historical control group [8] in which gastropexy was performed with two straight needles in 142 patients and the morbidity rate was 9.1% and the mortality was 0.7%. Most authors use device with two straight needles upon the performance of gastropexy [5,9,11,14,20] and described a morbidity ranging from 0 to 6.7% and a mortality rate varied from 0 to 2.9%. The results of our study support the premise that gastropexy performed with curved needles is a safe procedure. Gastropexy as presented here is a more simple option which is easy to perform and uses surgical suture material routinely available in the surgical room.

The technical variant presented here is also feasible because a high success index was obtained (92.4%). The majority of failure procedures were due to not reaching the gastric cavity with the curved needles, and these situations were solved with the use of straight needles as described in other study [8].

One limitation of the present study is that feasibility and safety were not evaluated in relation to a control group in which gastropexy would be performed with two straight needles. Another limitation is that the population studied was almost entirely composed of patients with malignant neoplasias and BMI < 30 kg/m^2 ^and the validity of the method in populations with neurological diseases and different BMI profiles needs to be evaluated. Another disadvantage of this new technical variant of gastropexy is that it can only be used in patients evaluated by endoscopy.

## Conclusion

The new gastropexy technical variant presented in this study has proven to be feasible and safe. This technique yielded low rates of peristomal infection and made unnecessary the use of prophylactic antibiotics.

## List of abbreviations

PEG: Percutaneous Endoscopic Gastrostomy; G-tube: gastric tube; BMI: Body Mass Index.

## Competing interests

The authors declare that they have no competing interests.

## Authors' contributions

PMOC conceived the study, participated in its coordination and prepared the manuscript. DMMC contributed to conception and design, acquisition, analysis and interpretation of data, drafted and revised the manuscript. MDT did epidemiological assistance in analysis and interpretation of data results and helped to write the manuscript. FHE contributed to conception and design, drafted and revised the manuscript. OMM contributed to conception and design, drafted and revised the manuscript. All authors read and approved the final manuscript.

## Pre-publication history

The pre-publication history for this paper can be accessed here:

http://www.biomedcentral.com/1471-230X/9/48/prepub

## Supplementary Material

Additional file 1 **New technical variant of gastropexy for percutaneous endoscopic gastrostomy**. Video containing the described procedure. Click here for file
